# Saccharomonopyrones A–C, New α-Pyrones from a Marine Sediment-Derived Bacterium *Saccharomonospora* sp. CNQ-490

**DOI:** 10.3390/md15080239

**Published:** 2017-08-01

**Authors:** Chae-Yoon Yim, Tu Cam Le, Tae Gu Lee, Inho Yang, Hansol Choi, Jusung Lee, Kyung-Yun Kang, Jin Sil Lee, Kyung-Min Lim, Sung-Tae Yee, Heonjoong Kang, Sang-Jip Nam, William Fenical

**Affiliations:** 1 Department of Chemistry and Nanoscience, Ewha Womans University, Seoul 03760, Korea; yimgenie@gmail.com (C.-Y.Y.); lecamtu5789@gmail.com (T.C.L.); dbluesee@gmail.com (I.Y.); hansol871231@gmail.com (H.C.); 2 School of Earth and Environmental Sciences, Seoul National University, NS-80, Seoul 08826, Korea; berong35@snu.ac.kr (T.G.L.); leejusung@snu.ac.kr (J.L.); 3 College of Pharmacy, Sunchon National University, Suncheon 57922, Korea; nms-kang@nate.com (K.-Y.K.); sungtae@sunchon.ac.kr (S.-T.Y.); 4 College of Pharmacy, Ewha Womans University, Seoul 03760, Korea; wlstlf113@nate.com (J.S.L.); kmlim@ewha.ac.kr (K.-M.L.); 5 Research Institute of Oceanography, Seoul National University, NS-80, Seoul 08826, Korea; 6 Center for Marine Biotechnology and Biomedicine, Scripps Institution of Oceanography, University of California-San Diego, La Jolla, CA 92093-0204, USA

**Keywords:** *Saccharomonospora* sp., marine natural products, α-pyrones

## Abstract

Intensive study of the organic extract of the marine-derived bacterium *Saccharomonospora* sp. CNQ-490 has yielded three new α-pyrones, saccharomonopyrones A–C (**1**–**3**). The chemical structures of these compounds were assigned from the interpretation of 1D, 2D NMR and mass spectrometry data. Saccharomonopyrone A (**1**) is the first α-pyrone microbial natural product bearing the ethyl-butyl ether chain in the molecule, while saccharomonopyrones B and C possess unusual 3-methyl and a 6-alkyl side-chain within a 3,4,5,6-tetrasubstituted α-pyrone moiety. Saccharomonopyrone A exhibited weak antioxidant activity using a cation radical scavenging activity assay with an IC_50_ value of 140 μM.

## 1. Introduction

**Figure 1:**
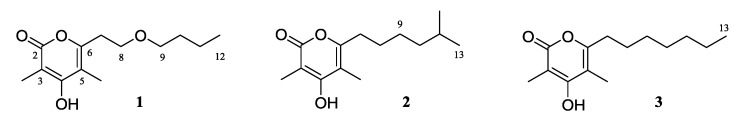
Structures of saccharomonopyrones A–C (**1**–**3**).

Actinobacteria are known as an abundant source of novel secondary metabolites comprising over 45% of all bioactive microbial metabolites known [1]. The recent discovery of numerous taxonomically unique marine actinomycetes, along with the isolation of structurally unprecedented secondary metabolites from these strains, illustrates marine actinomycetes as a promising source for the discovery of new natural products [2]. *Saccharomonospora*, a genus in the actinomycete family Pseudonocardiaceae, was first described in 1971 [3,4]. Members of the genus *Saccharomonospora* are interesting because they originate from diverse habitats and play an important role in the primary degradation of plant material by attacking hemicellulose [5]. Previous chemical investigations of members of this genus have led to the isolation of bioactive secondary metabolites, such as antibiotic AB 65, sakyomicin E, saccharonol A, antimicrobial saccharonol B, and piericidin A_3_ [6,7]. As part of our ongoing research for new secondary metabolites from marine actinobacteria, a *Saccharomonospora* bacterial strain CNQ-490 was documented. In a previous study, this strain was found to produce a novel cytotoxic alkaloid, lodopyridone A [8]. Further chemical investigation of this strain has now yielded three new natural products of the α-pyrone class. Herein, we report the isolation and structure elucidation of saccharomonopyrones A–C (**1**–**3**) along with their biological activities (Figure 1).

## 2. Results and Discussion

Saccharomonopyrone A (**1**) was obtained as a white amorphous powder. An high-resolution electrospray ionization mass spectrometry (HR-ESIMS) peak [M + H]^+^ at *m/z* 241.1446 (calcd for 241.1434) indicated the molecular formula C_13_H_20_O_4_, which implied four degrees of unsaturation. UV absorption at 288 nm and IR absorptions at 3402, 1672, and 1569 cm^−1^ indicated the presence of an α-pyrone moiety.

**Figure 2:**
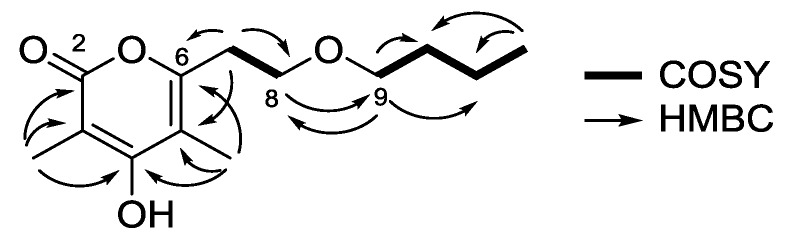
COSY and key HMBC correlations for saccharomonopyrone A (**1**).

The planar structure of **1** was confidently assigned from the interpretation of 1D and 2D NMR data. Analysis of the ^13^C NMR (Table 1) and heteronuclear single quantum correlation (HSQC) spectroscopic data identified three oxygenated quaternary sp^2^ carbons (δ_C_ 155.2, 164.3, and 164.5), two quaternary sp^2^ carbons (δ_C_ 97.3 and 107.5), along with two methyl singlets [3-CH_3_ (δ_H_ 1.82; δ_C_ 9.1) and 5-CH_3_ (δ_H_ 1.87; δ_C_ 9.9)], one methyl triplet (δ_H_ 0.83, *J* = 7.2 Hz; δ_C_ 13.6) and five methylene groups [CH_2_-7 (δ_H_ 2.70, *J* = 6.4 Hz; δ_C_ 31.0)/CH_2_-8 (δ_H_ 3.56, *J* = 6.4 Hz; δ_C_ 67.0)/CH_2_-9 (δ_H_ 3.35, *J* = 6.4 Hz; δ_C_ 69.6)/CH_2_-10 (δ_H_ 1.43, m; δ_C_ 31.1)/CH_2_-11 (δ_H_ 1.26, m; δ_C_ 18.7)]. Interpretation of ^1^H-^1^H correlation spectroscopy (COSY) correlations allowed the construction of two spin systems: [H-7 (δ_H_ 2.70)/H-8 (δ_H_ 3.56)] and [H-9 (δ_H_ 3.35)/H-10 (δ_H_ 1.43)/H-11 (δ_H_ 1.26)/H-12 (δ_H_ 0.83)]. Heteronuclear multiple bond correlation (HMBC) correlations from H-8 to C-9 (δ_C_ 69.6) and from H-9 to C-8 (δ_C_ 67.0), as well as the oxygenated carbon chemical shifts of C-8 and C-9, supported the attachment of two spin systems through an oxygen atom (Figure 2).

The α-pyrone functionality was constructed as 3,5-dimethyl-4-hydroxypyran-2-one with a side chain substituent at C-6 by analysis of ^13^C chemical shifts and HMBC correlations. HMBC correlations from 3-CH_3_ to C-2 (δ_C_ 164.5), C-3 (δ_C_ 97.3), C-4 (δ_C_ 164.3), and from 5-CH_3_ to C-4, C-5 (δ_C_ 107.5), C-6 (δ_C_ 155.2), indicated the position of the methyl groups on the α-pyrone ring. Lastly, the HMBC cross-peaks from H-7 to C-6 and C-5 supported the connectivity of the α-pyrone ring to the linear chain, thus completing the structure assignment of saccharomonopyrone A (**1**).

Saccharomonopyrone B (**2**) was isolated as an amorphous white powder. Its molecular formula C_14_H_22_O_3_ was determined by the observation of a HR-ESIMS ion peak [M + H]^+^ at *m/z* 239.1651, (calcd for 239.1642). The infrared (IR) absorption at 1673 cm^−1^ was assigned to a conjugated ketone functionality. The ^1^H NMR spectrum of **2** was similar to that of **1** except for the absence of two oxygenated methylene protons and the presence of two methyl doublets. COSY NMR correlations (H-7 (δ_H_ 2.47)/H-8 (δ_H_ 1.51)/H-9 (δ_H_ 1.29)/H-10 (δ_H_ 1.17), H-12 (δ_H_ 0.85)/H-11 (δ_H_ 1.51)/H-13 (δ_H_ 0.85)) and HMBC correlations of H-12 and H-13 to C-10 (δ_C_ 38.4), and from H-9 (δ_H_ 1.29) to C-10, and from H-10 to C-9 (δ_C_ 26.6) permitted the construction of the aliphatic chain as a 5-methylhexyl moiety. The linkage of the alkyl moiety to the α-pyrone ring was secured from the HMBC correlation from H-7 to C-6 (δ_C_ 158.2), completing the structure assignment of saccharomonopyrone B (**2**) as 4-hydroxy-3,5-dimethyl-6-(5-methylhexyl)-α-pyrone.

Saccharomonopyrone C (**3**) was also obtained as a white amorphous powder. The formula of **3** was deduced as C_14_H_22_O_3_ by the observation of the [M + H]^+^ ion peak at *m/z* 239.1665 (calcd for 239.1642). Compound **3** had the same molecular formula as **2**. The only difference was that the ^1^H NMR of **3** displayed a methyl triplet. The analysis of 2D NMR spectroscopic data allowed the establishment of **3** as 4-hydroxy-3,5-dimethyl-6-heptyl-α-pyrone.

Pyrones are a well-known class of microbial secondary metabolites and are found to have a wide range of biological activities such as anti-inflammatory [9], anticancer [10], antimicrobial [11,12], anti-obesity [13,14], and antibiotic activities [15]. Previous studies on microbial sources have shown that the main producer of α-pyrones is fungi, but α-pyrones are also produced by plants, animals, and bacteria. Marine bacteria have also yielded α-pyrones with interesting bioactivities, examples of which are antibiotic Sch419560 [15], germicidins, and cytotoxic violapyrones. A recent study also expanded our knowledge for the first time that pyrones can be used as signaling molecules of the bacterial cell-cell communications in the soil bacterium *Pseudomonas* [16]. Bacteria monitor other bacteria in their living environment by producing and responding to signaling molecules. This led to a strategy to prevent pathogenicity by blocking bacterial communication in their environment [17,18,19].

Saccharomonopyrones A–C (**1**–**3**) were tested on various assays such as monoamine oxidase (MAO) inhibitory, acetylcholinesterase (AChE) inhibitory, β-site amyloid precursor protein cleaving enzyme 1 (BACE1), anti-osteoporosis, cytotoxicity, anti-tyrosinase, and antibacterial activities. However, the compounds did not display any significant biological activities in these assays. Interestingly, saccharomonopyrone A (**1**) showed weak antioxidant activity measuring free radical scavenging activity and cation radical scavenging activity in assays with IC_50_ values of 911 μM and 140 μM, respectively. 

Saccharomonopyrone A (**1**) has an unusual ethylbutyl ether moiety attached at C-6. There are no reports of an ether moiety attached at C-6 within the α-pyrone class. Some similar molecules with ethyl-methyl sulfide and propyl-methyl sulfide groups were obtained as bioengineered products [20]. A similar compound possessing an ethyl chain with an acetyl end group was obtained during the synthesis of β-polyketones [21]. In addition, another naturally occurring and structurally similar compound is known to possess a propyl chain with a methyl carbonyl end attached at the C-6 position [22]. However, the butoxyethyl side chain in **1** is unprecedented within this class of natural products. 

Nocapyrone R and violapyrone B are the most structurally related compounds of saccharomonopyrone B (**2**) [11,23]. The only difference is that nocapyrone R has a methoxy group at C-3 and violapyrone B has no methyl group on C-5. Violapyrone I, structurally the most similar natural product of saccharomonopyrone C (**3**), also lacks the methyl group on C-5 [10].

The genus *Saccharomonospora* (Family Pseudonocardiaceae), with eleven known species to date, has been known since 1971 [24]. *Saccharomonospora* sp. CNQ-490 is a unique sediment-derived strain which produces the structurally unprecedented lodopyridones A–C [8,25]. Furthermore, by genome mining, this strain has been shown to possess a full biosynthetic pathway to produce a new antibiotic taromycin A through direct cloning and refactoring methods [26]. Saccharomonopyrones A–C, and previously reported natural products, lodopyridones and taromycin A, illustrate the versatile secondary metabolites producing ability of this strain. The application of a recent genetic modification method for the biosynthetic gene cluster of this strain to introduce a methyl group in natural products could lead to the successful production of new secondary metabolites [27].

## 3. Materials and Methods

### 3.1. General Experimental Procedures

The UV spectra were recorded in methanol (MeOH) on a UVS-2100 (Scinco, Seoul, Korea). The IR spectra were obtained using a Varian Scimitar Series. The NMR spectra were obtained using a Varian Inova NMR spectrometer (500 and 125 MHz for ^1^H and ^13^C NMR, respectively, Varian Inc., Palo Alto, CA, USA), using the signals of the residual solvent as internal references and δ_H_ 2.50 and δ_C_ 39.5 ppm for dimethyl sulfoxide-*d*_6_ (DMSO). High Resolution Mass spectra were determined by a JMS-AX505WA mass spectrometer (Jeol Ltd., Tokyo, Japan). Low-resolution LC-MS data were analyzed using an Agilent Technologies 6120 quadrupole LC/MS system with a reversed-phase column (Phenomenex Luna C_18_(2) 100 Å, 50 mm × 4.6 mm, 5 μm, Phenomenex, Torrance, CA, USA) at a flow rate of 1.0 mL/min (Agilent Technologies, Santa Clara, CA, USA). Column chromatographic separation was performed using C_18_ (40–63 μm, ZEOprep 90, Zeochem, Zurich, Switzerland) with a gradient solvent of MeOH and water (H_2_O). The fractions were purified using reversed-phase HPLC (Waters 600S controller with 996 PDA detector (Waters Corporation, Milford, MA, USA), Phenomenex Luna C_18_ (250 mm × 10 mm, 5 μm) column (Phenomenex, Torrance, CA, USA)) with a mixture of acetonitrile (ACN) and H_2_O at flow rate of 2.0 mL/min. All chemicals were purchased from Sigma-Aldrich (Sigma-Aldrich, St. Louis, MO, USA) and used without further purification.

### 3.2. Strain Isolation and Fermentation

Actinomycete strain *Saccharomonospora* sp. CNQ-490 was collected from a deep sea sediment sample 2 km west of the Scripps pier, in La Jolla, CA, USA. The 16S rDNA sequence of this strain showed modest identity with the genus *Saccharomonospora* (accession number: KF301601). Strain CNQ-490 was cultured in 40-L scale using 2.5-L Ultra Yield (Thomson Instrument Company, Oceanside, CA, USA) flasks, each containing 1 L of the medium (10 g/L of soluble starch, 2 g/L of yeast, 4 g/L of peptone, 10 g/L of CaCO_3_, 20 g/L of KBr, 8 g/L of Fe_2_(SO_4_)_3_·4H_2_O dissolved in 1000 mL artificial seawater) at 25 °C with shaking at 150 rpm. After 7 days, the broth was extracted with ethyl acetate (added ratio 1:1 of volume). The ethyl acetate layer was separated and dried with anhydrous sodium sulfate. The organic solvent was removed to yield 2.5 g of the organic extract.

### 3.3. Extraction and Isolation

The crude organic extract was loaded on the C_18_ resin and fractionated by reversed-phase C_18_ (40–63 μm, ZEO prep 90) flash chromatography with gradient elution (from 80% of H_2_O in MeOH to 100% MeOH) to yield eight fractions. Fractions five and six eluted with 40% and 30% of H_2_O, respectively, were further purified by reversed-phase HPLC (Phenomenex Luna C_18_ column 250 mm × 10 mm, 5 μm) under isocratic conditions (50% of ACN in H_2_O) to give 5.7 mg of saccharomonopyrone A (**1**) (retention time 15.4 min), along with saccharomonopyrones B (**2**) (6.7 mg, retention time 45.6 min) and C (**3**) (4.9 mg, retention time 49.6 min).

Saccharomonopyrone A (**1**): amorphous white solid, UV (MeOH) λ_max_ (log ε) 209 (2.53), 288 (2.49) nm; IR (KBr) ν_max_ 3402, 2941, 1672, 1569 cm^−1^, ^1^H and ^13^C NMR data, Table 1 and Supplementary Materials; HRESIMS *m/z* 241.1446 [M + H]^+^ (calcd for C_13_H_21_O_4_, 241.1434).

Saccharomonopyrone B (**2**): amorphous white solid, UV (MeOH) λ_max_ (log ε) 203 (2.53), 292 (2.32) nm; IR (KBr) ν_max_ 3427, 2940, 1673, 1575 cm^−1^, ^1^H and ^13^C NMR data, Table 1 and Supplementary Materials; HRESIMS *m/z* 239.1651 [M + H]^+^ (calcd for C_14_H_23_O_3_, 239.1642).

Saccharomonopyrone C (**3**): amorphous white solid, UV (MeOH) λ_max_ (log ε) 201 (2.53), 291 (2.20) nm; IR (KBr) ν_max_ 3395, 2930, 1665, 1560 cm^−1^, ^1^H and ^13^C NMR data, Table 1 and Supplementary Materials; HRESIMS *m/z* 239.1665 [M + H]^+^ (calcd for C_14_H_23_O_3_, 239.1642).

### 3.4. Bioactivity Assays

MAO inhibitory assay [28], AChE inhibitory assay [29], BACE1 [25], anti-osteoporosis assay [30], and anti-tyrosinase assay [31] were performed following the previously published methods. Cytotoxicity tests were performed on two human kidney cancer cell lines, A498 and ACHN renal cancers, according to previously published methods [32]. Antibacterial assays were performed against seven bacterial strains including four Gram-positive (*Staphylococcus epidermidis* ATCC 12228, *Kocuria rhizophila* ATCC 9341, *Bacillus subtilis* ATCC 6633, *Staphylococcus aureus* ATCC 65381) and three Gram-negative (*Escherichia coli* ATCC 11775, *Salmonella typhimurium* ATCC 14028, *Klebsiella pneumoniae* ATCC 4352) strains following a previously published method [25]. The antioxidant activity was performed using the 1,1-diphenyl-2-picrylhydrazyl free radical (DPPH) as described previously with slight modification [33]. The DPPH solution (0.45 mM) was prepared daily in a 20-mL conical tube and kept in the dark at 4 °C. The DPPH solution (120 µL) was added to 60 µL of sample, control, or standard solution in 70% ethanol at different concentrations. The solutions were mixed, covered, and allowed to react in the dark for 15 min; afterward, the absorbance at 517 nm was read. Ascorbic acid was used as a positive control (IC_50_ 21.02 ± 0.82 µM). Data are presented as the mean values ± standard deviation (SD) of three measurements. The free radical scavenging activity of each solution was then calculated as the percent inhibition according to the following equation:

Scavenging rate (%) = [A (blank) − A (sample)]/A (blank) × 100

2,2′-Azino-bis(3-ethylbenzothiazoline-6-sulfonic acid) diammonium salt (ABTS) cation radical scavenging activity of the compounds were tested using the spectroscopic method described by Roberta et al. [34]. The ABTS cation radical (ABTS+) was acquired by reacting 7 mM solution of ABTS with 2.45 mM potassium persulfate reaction. The mixture was left to stand in the dark at room temperature for 12–16 h before use. Prior to the assay, the ABTS radical cation solution was diluted with ethyl alcohol to an absorbance of 0.750 ± 0.05 at 734 nm. The ABTS+ solution was then added to each sample, standard, and control solution. Ascorbic acid was used as a positive control (IC_50_ 13.01 ± 0.21 µM). Data are presented as the mean values ± standard deviation (SD) of the three measurements. The extent of decolorization is calculated as a percent reduction in absorbance. The percentage of cation radical scavenging was computed using the following equation:

Scavenging rate (%) = [A (blank) − A (sample)]/A (blank) × 100

### 3.5. Statistical Analyses

Statistical analyses for DPPH and ABTS cation radical scavenging activities were performed using GraphPad Prism 5 (GraphPad Software, Inc., La Jolla, CA, USA). The nonlinear regression was used to determine the dose-response inhibition. Results are expressed as means ± standard deviation of three independent experiments.

## 4. Conclusions

Three new α-pyrones, saccharomonopyrones A–C (**1**–**3**), were isolated from the marine sediment-derived bacterium *Saccharomonospora* sp. strain CNQ-490. Saccharomonopyrones A–C (**1**–**3**) are 3,4,5,6-tetra-subtituted α-pyrones. Saccharomonopyrone A is unusual in having an ether moiety attached at C-6, observed for the first time in this class of natural products. Analysis of the tetra-substituted α-pyrone biosynthetic gene cluster from this strain may provide an opportunity to discover diverse α-pyrone analogues.

## Figures and Tables

**Table 1:** ^1^H and ^13^C NMR spectral data for saccharomonopyrones A–C (**1**–**3**) in DMSO-*d*_6_^1^.

No. C	(1)		(2)		(3)	
	δ_C_, Type	δ_H_, Mult. ^2^ (*J* in Hz)	δ_C_, Type	δ_H_, Mult. ^2^ (*J* in Hz)	δ_C_, Type	δ_H_, Mult. ^2^ (*J* in Hz)
**2**	164.5		165.1		164.6	
**3**	97.3		97.6		97.1	
**4**	164.3		164.9		164.4	
**5**	107.5		107.0		106.5	
**6**	155.2		158.2		157.8	
**7**	31.0, CH_2_	2.70, t (6.4)	30.4, CH_2_	2.47, t (6.5)	29.9, CH_2_	2.45, t (7.5)
**8**	67.0, CH_2_	3.56. t (6.4)	27.6, CH_2_	1.51, m ^3^	26.8, CH_2_	1.51, m
**9**	69.6, CH_2_	3.35, t (6.4)	26.6, CH_2_	1.29, m	28.3, CH_2_	1.26, m ^3^
**10**	31.1, CH_2_	1.43, m	38.4, CH_2_	1.17, m	28.3, CH_2_	1.26, m ^3^
**11**	18.7, CH_2_	1.26, m	27.8, CH	1.51, m^3^	31.1, CH_2_	1.25, m ^3^
**12**	13.6, CH_3_	0.83, t (7.2)	22.9, CH_3_	0.85, d (6.6)	22.0, CH_2_	1.26, m ^3^
**13**			22.9, CH_3_	0.85, d (6.6)	13.8, CH_3_	0.85, t (7.1)
**3-CH_3_**	9.1, CH_3_	1.82, s	9.6, CH_3_	1.83, s	9.1, CH_3_	1.82, s
**5-CH_3_**	9.9, CH_3_	1.87, s	10.3, CH_3_	1.88, s	9.8, CH_3_	1.87, s

^1^ 300 MHz for ^1^H NMR, 75 MHz for ^13^C NMR. ^2^ Number of attached protons was deduced from 2D NMR analysis. ^3^ Signals were overlapping.

## Supplementary Materials

The following are available online at www.mdpi.com/1660-3397/15/8/239/s1; ^1^H, ^13^C and 2D NMR spectroscopic data of saccharomonopyrones A–C (**1**–**3**).
