# Supplementary material for: Saccharomonopyrones A–C, New α-Pyrones from a Marine Sediment-Derived Bacterium Saccharomonospora sp. CNQ-490
Source: Mar Drugs. 2017 Aug 1;15(8):239. doi: 10.3390/md15080239 (PMC5577594; doi:10.3390/md15080239)
Supplement: Supplementary file 1 [file marinedrugs-15-00239-s001.pdf]

Supporting Information for

Saccharomonopyrones A - C, New α-Pyrones from a Marine Sediment-Derived Bacterium *Saccharomonospora* sp. CNQ-490

Chae-Yoon Yim [1,†], Tu Cam Le [1,†], Tae Gu Lee [2,†], Inho Yang [1], Hansol Choi [1], Jusung Lee [2], Kyung-Yun Kang [3], Jin Sil Lee [4], Kyung-Min Lim [4], Sung-Tae Yee [3], Heonjoong Kang [2,5,*], Sang-Jip Nam [1,*] and William Fenical [6,*]

[1] Department of Chemistry and Nanoscience, Ewha Womans University, Seoul 03760, Republic of Korea
[2] School of Earth and Environmental Sciences, Seoul National University, NS-80, Seoul 08826, Republic of Korea
[3] College of Pharmacy, Sunchon National University, Suncheon, 57922, Republic of Korea
[4] College of Pharmacy, Ewha Womans University, Seoul 03760, Republic of Korea
[5] Research Institute of Oceanography, Seoul National University, NS-80, Seoul 08826, Republic of Korea
[6] Center for Marine Biotechnology and Biomedicine, Scripps Institution of Oceanography, University of California-San Diego, La Jolla, CA 92093-0204, USA
* Correspondence: hjkang@snu.ac.kr; Tel.: +81-2-880-5730 (H.K.) sjnam@ewha.ac.kr; Tel.: +82-10-5210-7715 (S.N.) wfenical@ucsd.edu; Tel: +1-858-259-3839 (W.F.)

Table of Contents

Figure S1. ^{1}H NMR Spectrum (300 MHz) of saccharomonopyrone A (**1**) in DMSO-d_6…………………….......... S3

Figure S2. ^{13}C NMR Spectrum (75 MHz) of saccharomonopyrone A (**1**) in DMSO-d_6………………………….. S4

Figure S3. gCOSY Spectra (500 MHz) of saccharomonopyrone A (**1**) in DMSO -d_6… …………………………. S5

Figure S4. gHSQC Spectra (500 MHz) of saccharomonopyrone A (**1**) in DMSO -d_6…………………………….. S6

Figure S5. gHMBC Spectra (500 MHz) of saccharomonopyrone A (**1**) in DMSO -d_6……………………………. S7

Figure S6. ^{1}H NMR Spectrum (300 MHz) of of saccharomonopyrone B (**2**) in DMSO -d_6………………………. S8

Figure S7. ^{13}C NMR Spectrum (75 MHz) of saccharomonopyrone B (**2**) in DMSO -d_6……………..……….... S9

Figure S8. gCOSY Spectra (500 MHz) of saccharomonopyrone A (**2**) in DMSO -d_6.. S10

Figure S9. gHSQC Spectra (500 MHz) of saccharomonopyrone A (**2**) in DMSO -d_6.. S11

Figure S10. gHMBC Spectra (500 MHz) of saccharomonopyrone A (**2**) in DMSO -d_6 .... S12

Figure S11. ^{1}H NMR Spectrum (300 MHz) of saccharomonopyrone C (**3**) in DMSO -d_6……………….................. S13

Figure S12. ^{13}C NMR Spectrum (75 MHz) of saccharomonopyrone C (**3**) in DMSO -d_6………………………… S14

Figure S13. gCOSY Spectra (500 MHz) of saccharomonopyrone A (**3**) in DMSO -d_6.. S15

Figure S14. gHSQC Spectra (500 MHz) of saccharomonopyrone A (**3**) in DMSO -d_6.. S16

Figure S15. gHMBC Spectra (500 MHz) of saccharomonopyrone A (**3**) in DMSO -d_6 .... S17

Figure S16. UV spectra of Saccharomonopyrones……………………………………………………………. S18

Figure S1. ^{1}H NMR Spectrum (300 MHz) of Saccharomonopyrone A (**1**) in DMSO-d_6

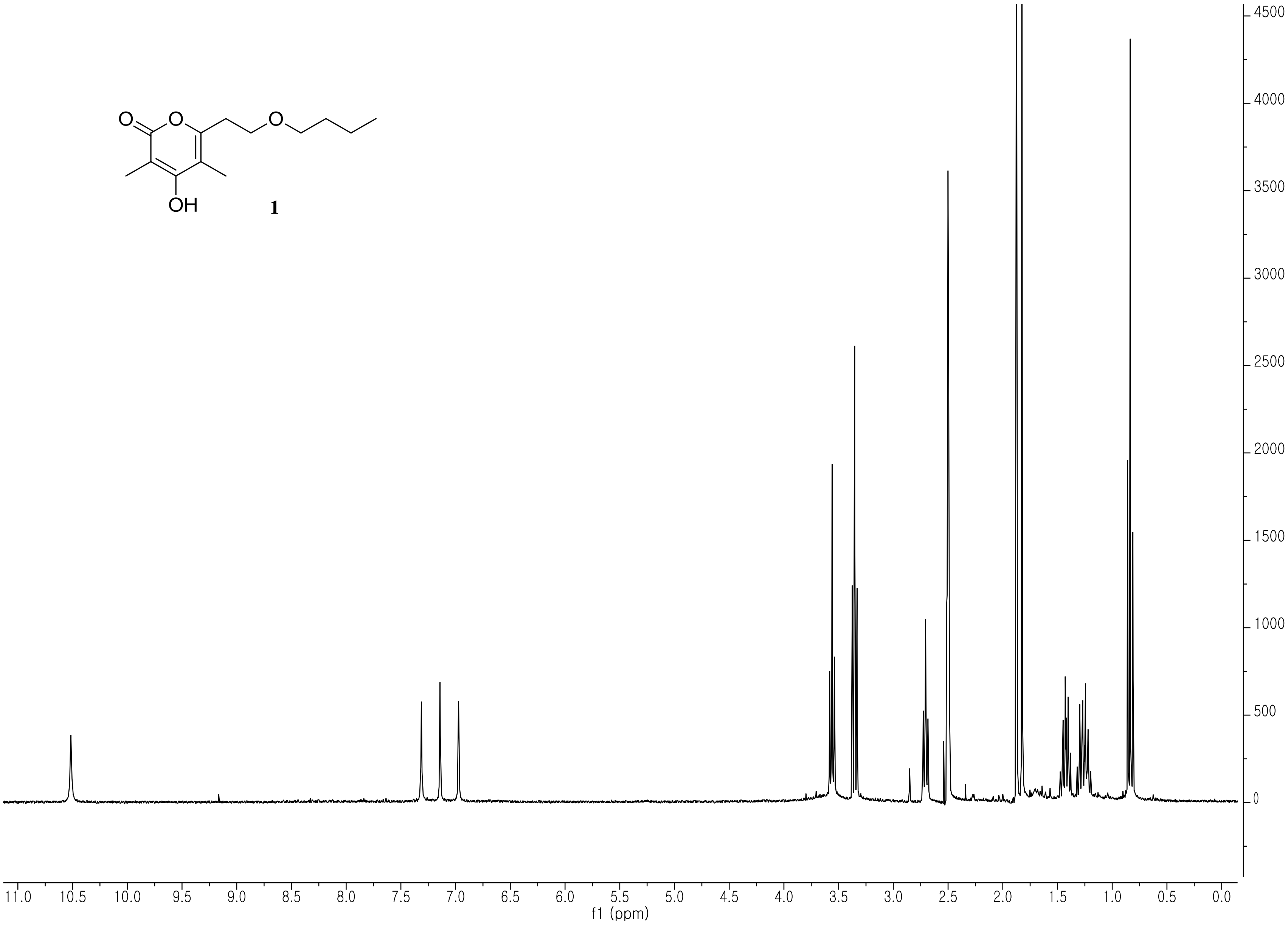

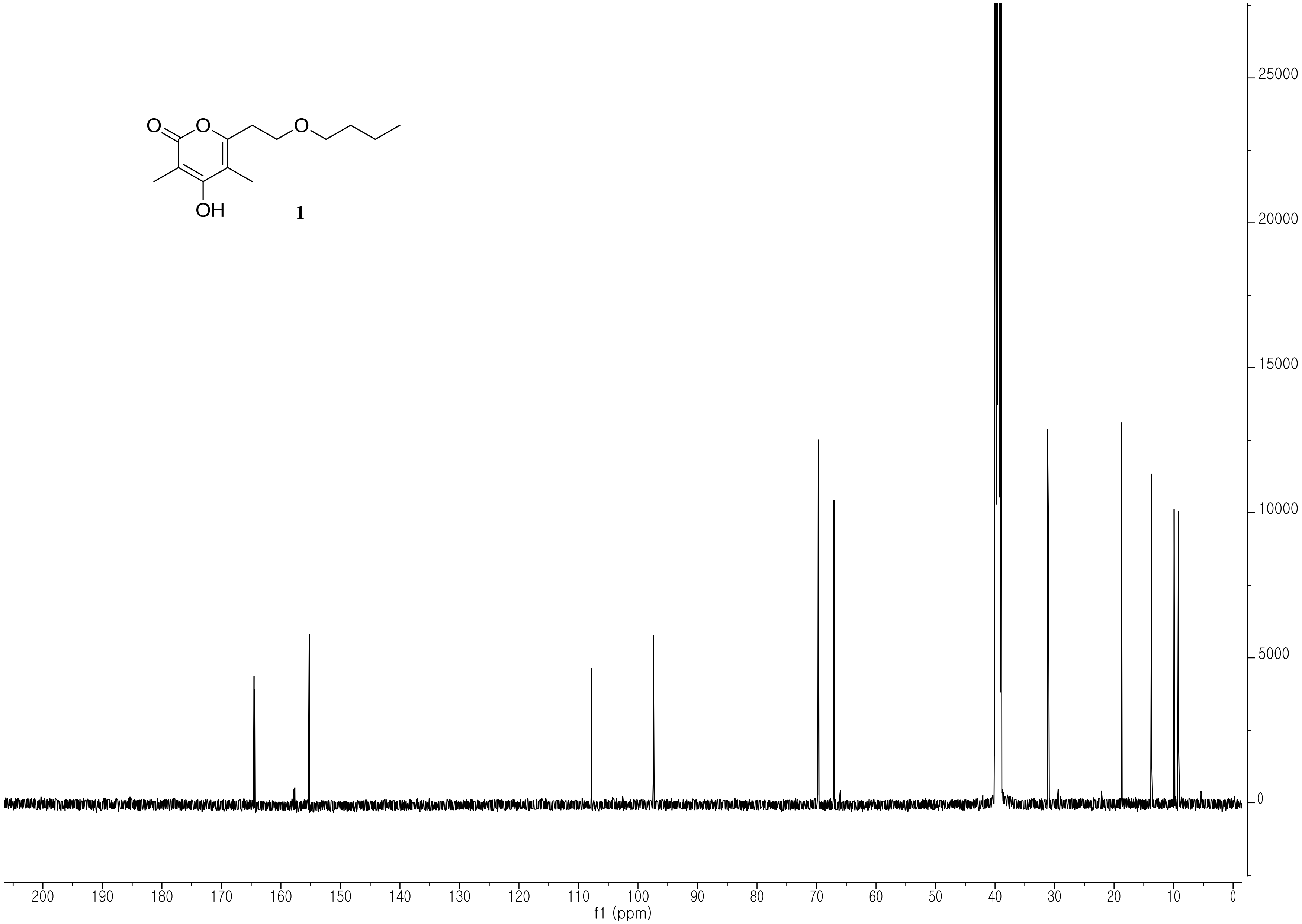

O
O
O
OH
1
25000
20000
15000
10000
5000
0
200 190 180 170 160 150 140 130 120 110 100 90 80 70 60 50 40 30 20 10 0
f1 (ppm)

Figure S3. gCOSY Spectra (500 MHz) of Saccharomonopyrone A (**1**) in DMSO-d_6

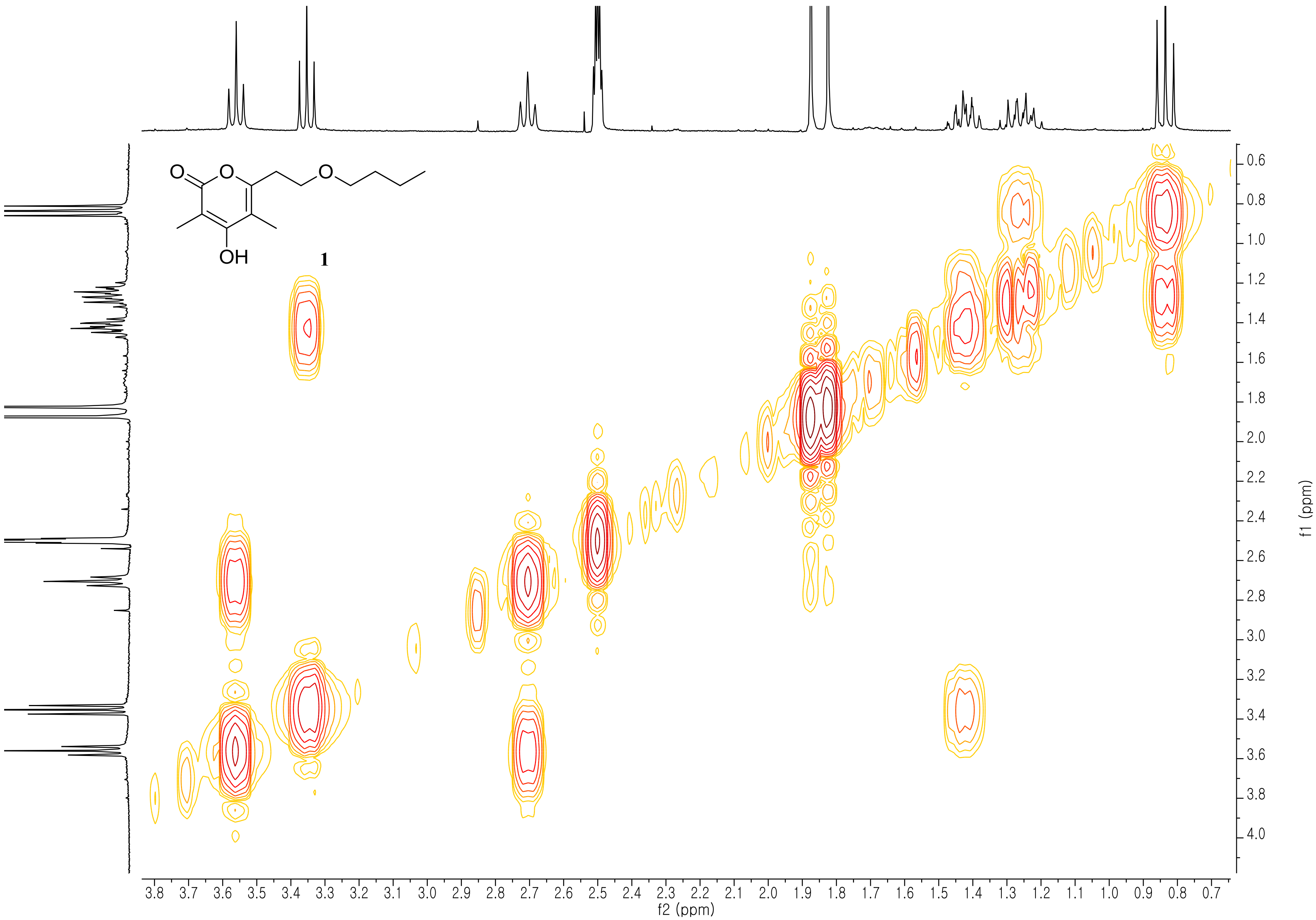

Figure S4. gHSQC Spectra (500 MHz) of Saccharomonopyrone A (**1**) in DMSO-d_6

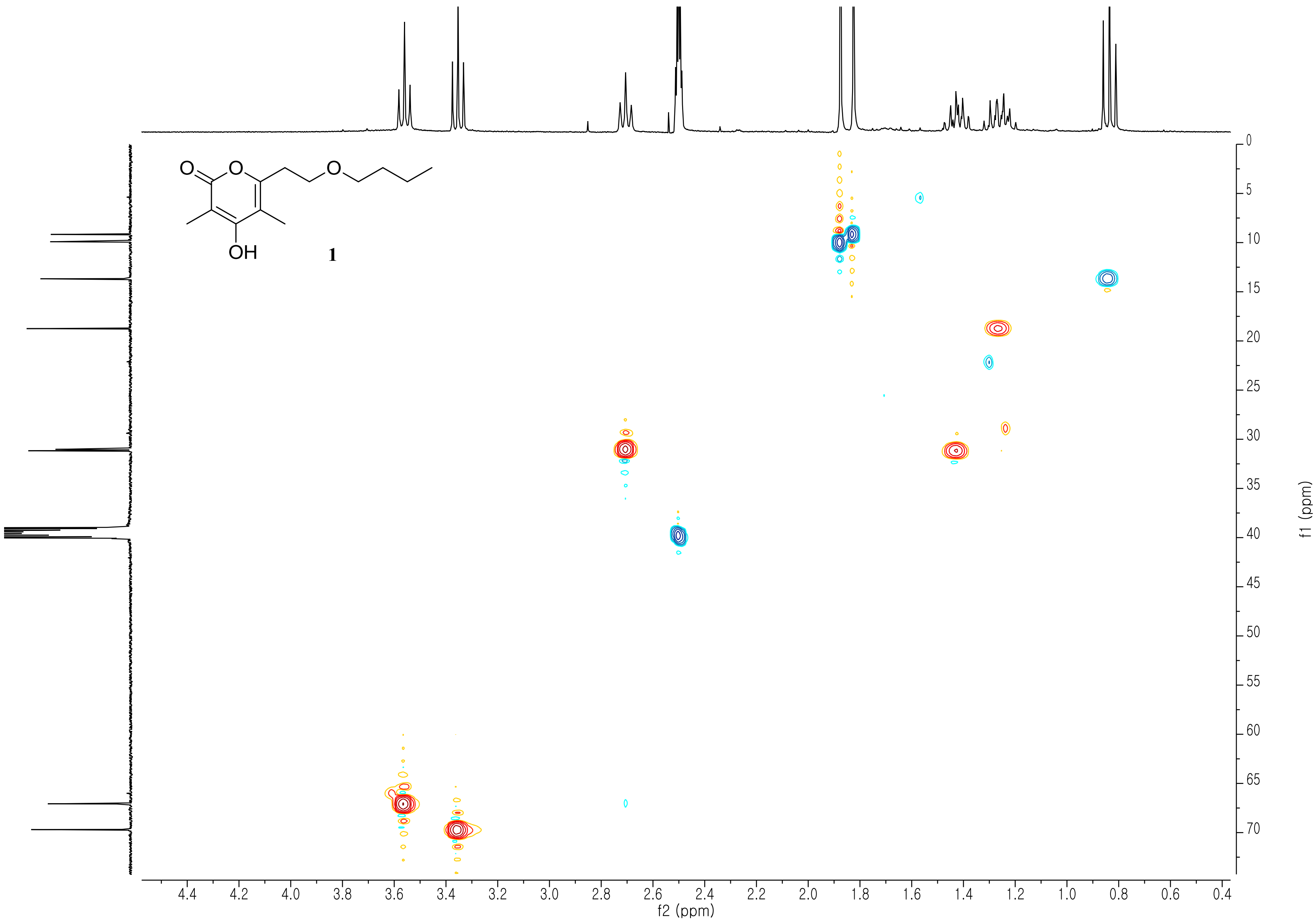

Figure S5. gHMBC Spectra (500 MHz) of Saccharomonopyrone A (**1**) in DMSO-d_6

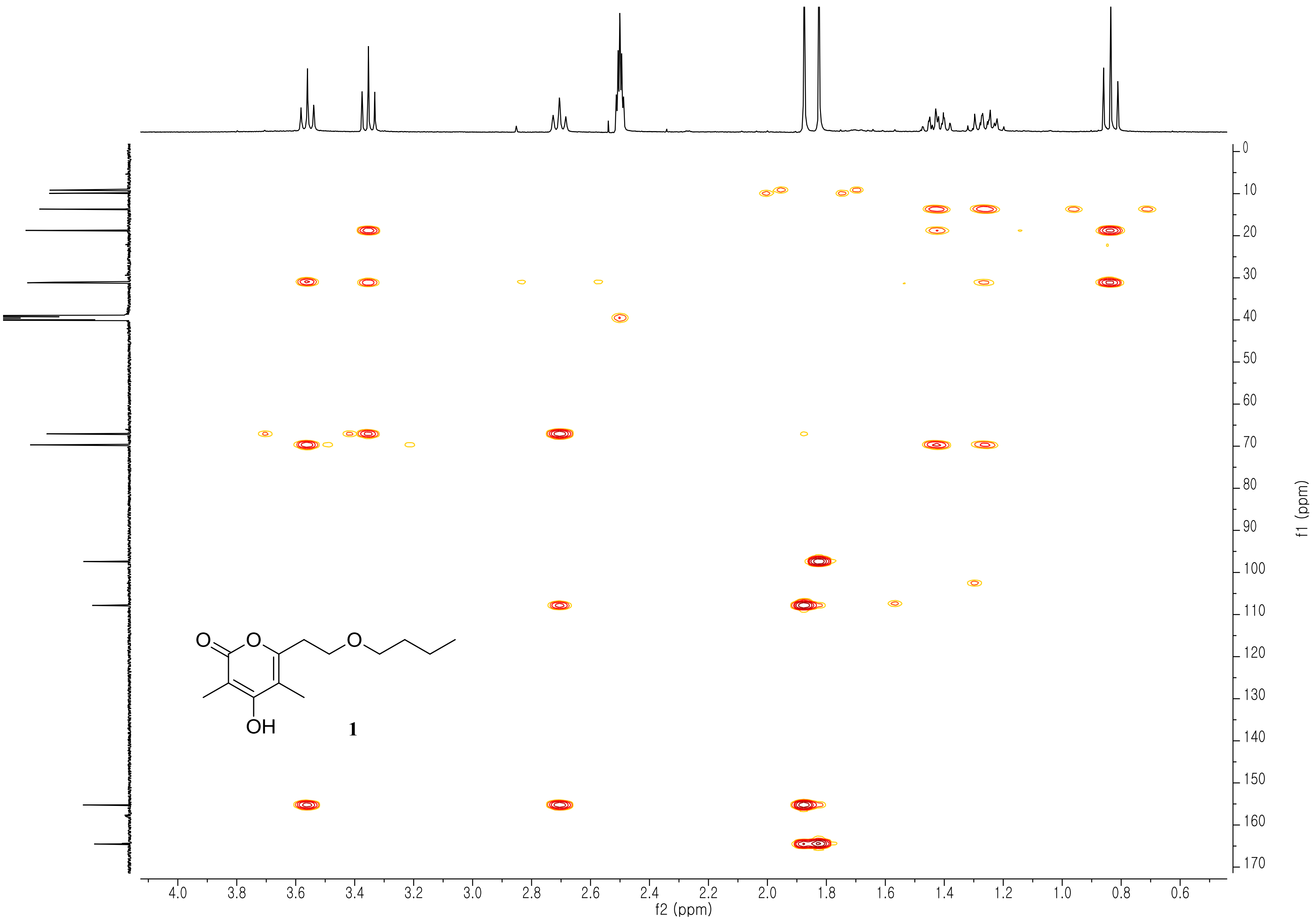

Figure S6. ^{1}H NMR Spectrum (300 MHz) of Saccharomonopyrone B (**2**) in DMSO-d_6

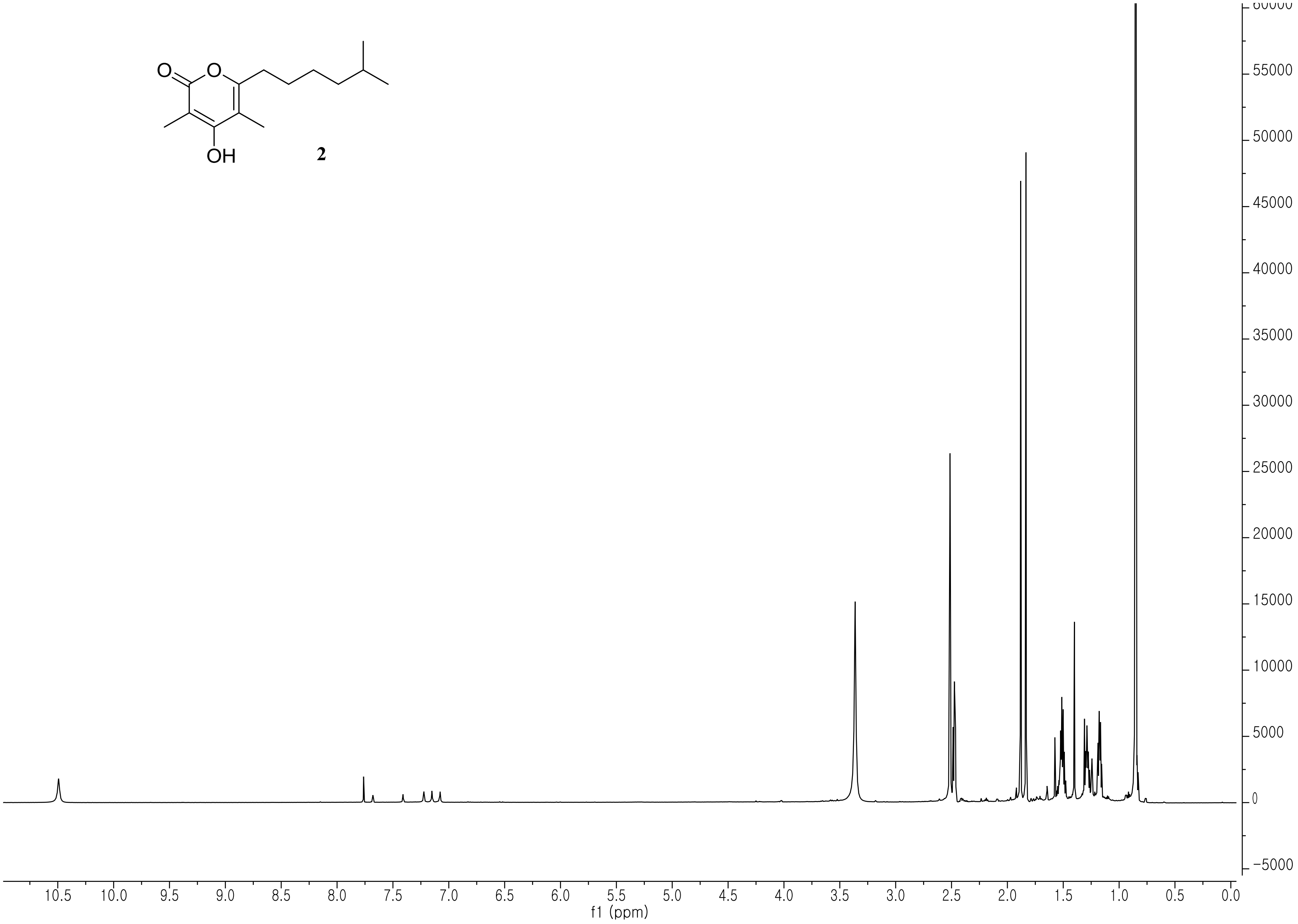

Figure S7. ^{13}C NMR Spectrum (75 MHz) of Saccharomonopyrone B (**2**) in DMSO-d_6

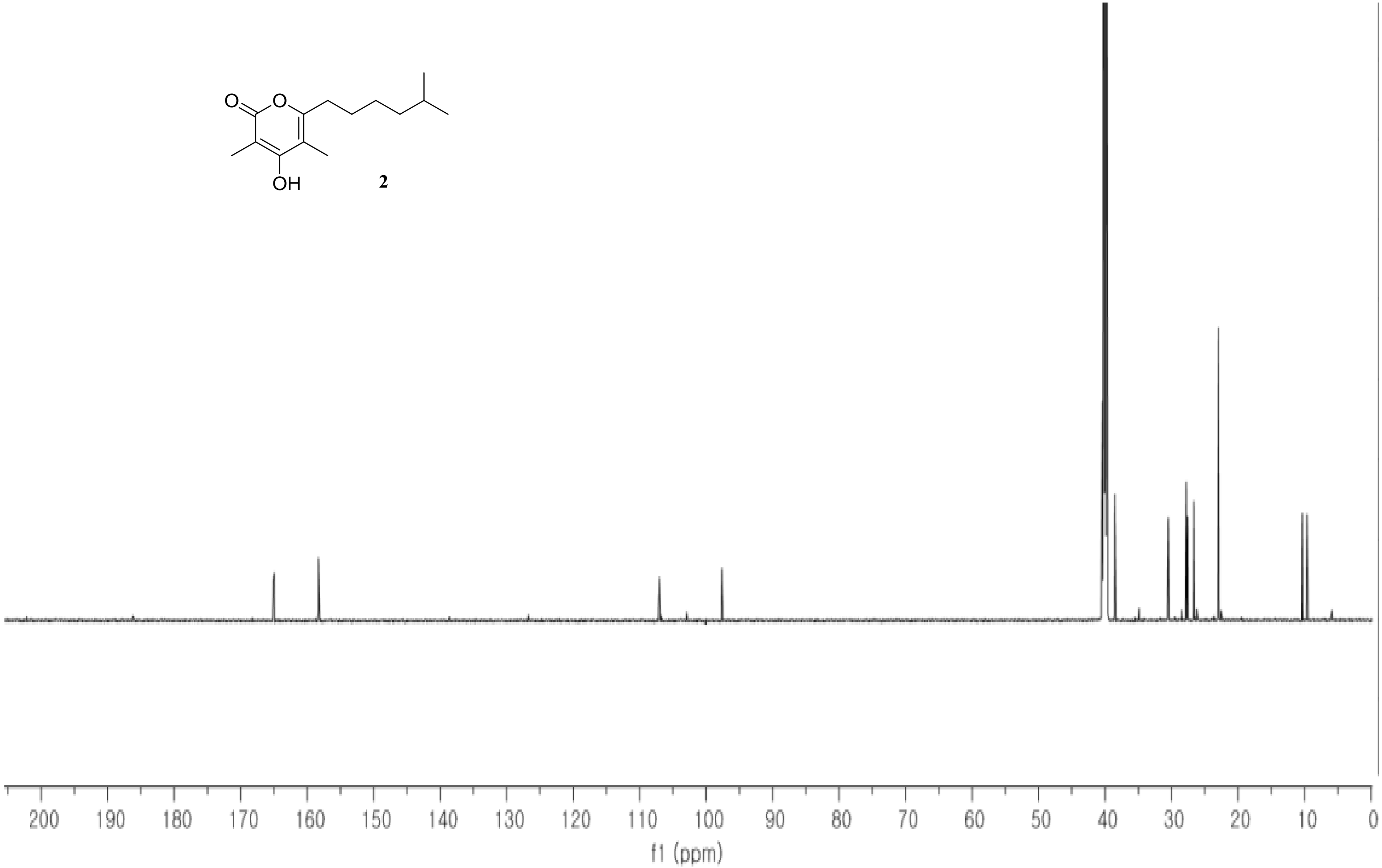

Figure S8. gCOSY Spectra (500 MHz) of Saccharomonopyrone A (**2**) in DMSO-d_6

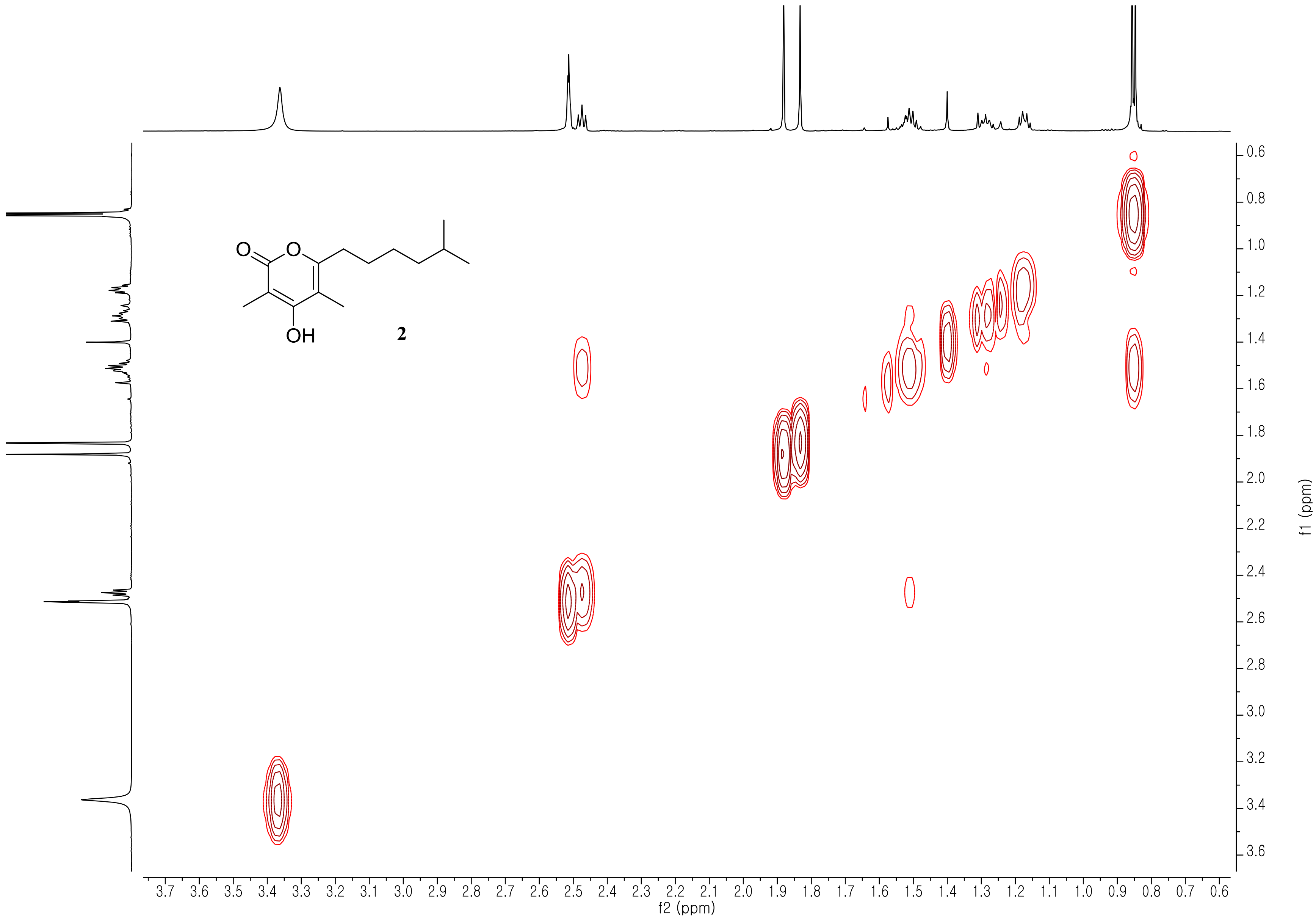

Figure S9. gHSQC Spectra (500 MHz) of Saccharomonopyrone A (**2**) in DMSO-d_6

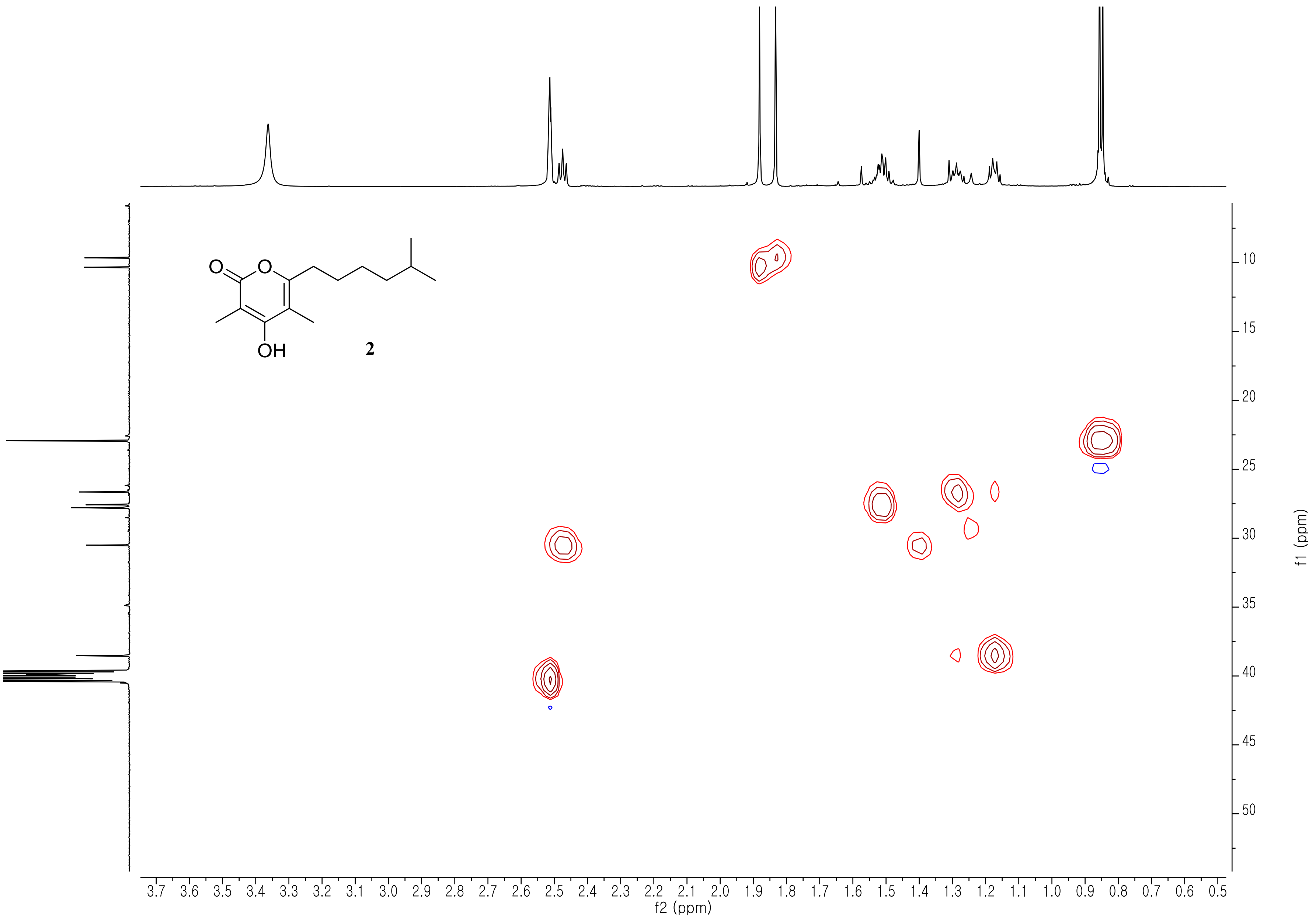

Figure S10. gHMBC Spectra (500 MHz) of Saccharomonopyrone A (**2**) in DMSO-d_6

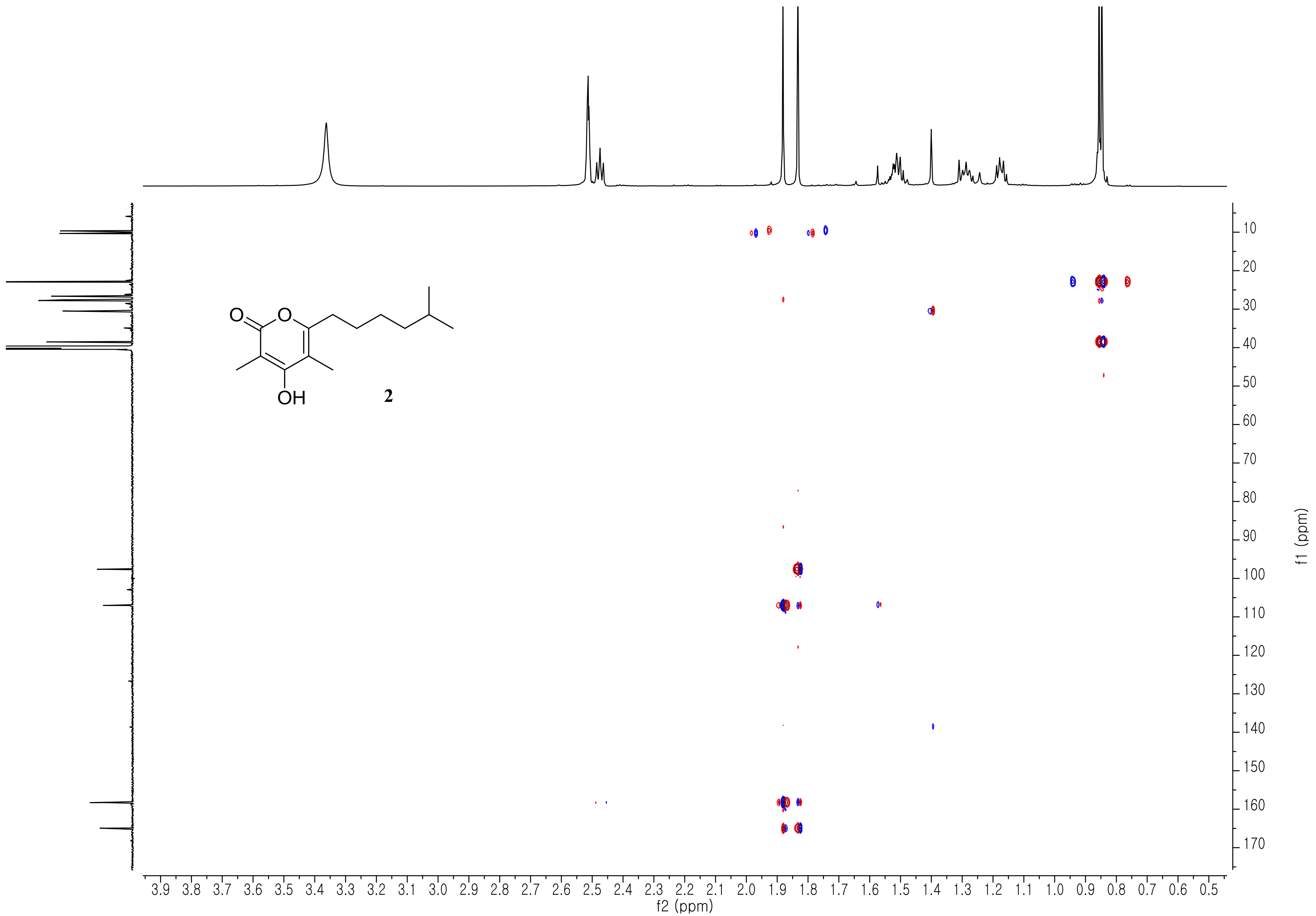

Figure S11. ^{1}H NMR Spectrum (300 MHz) of Saccharomonopyrone C (**3**) in DMSO-d_6

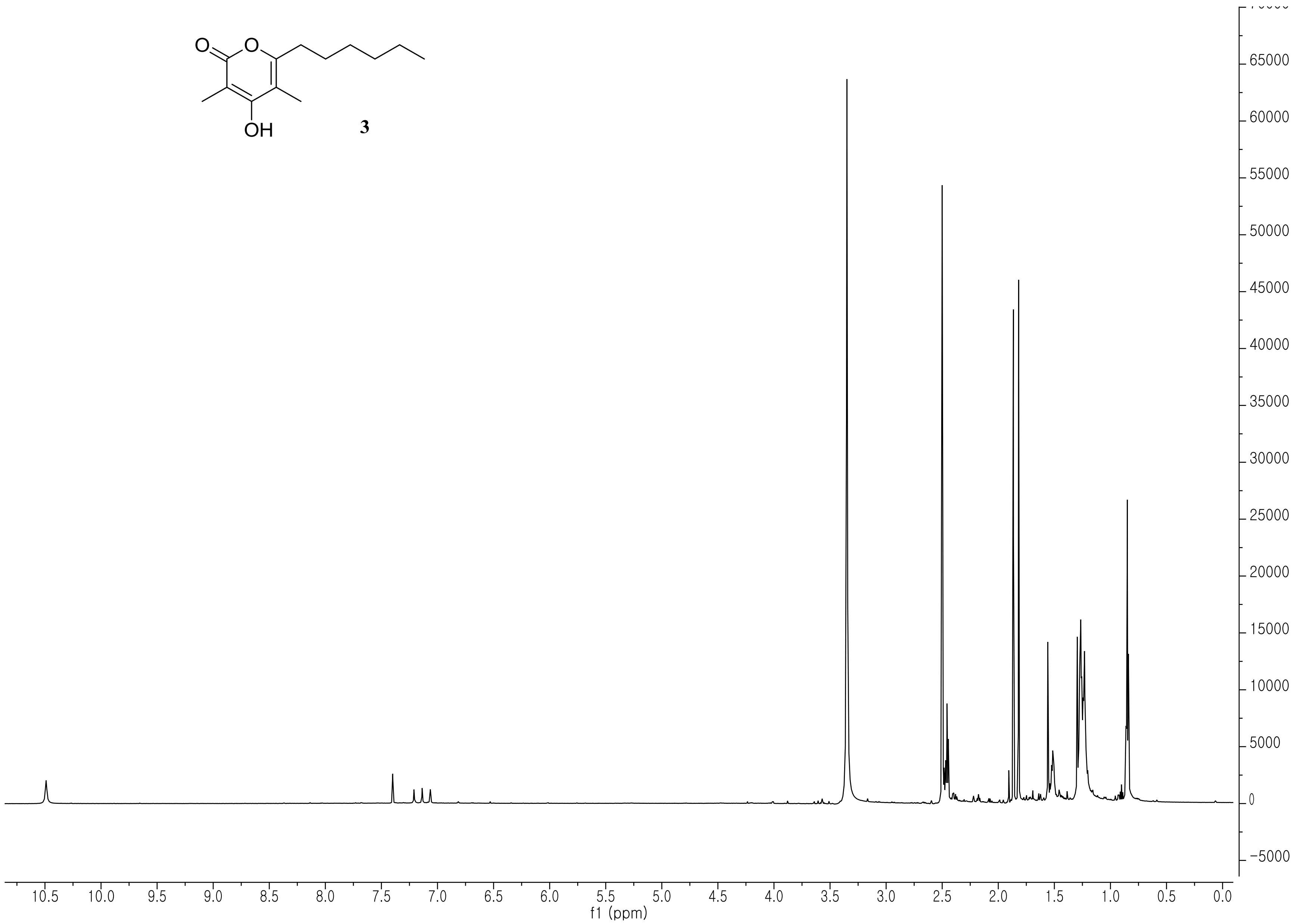

Figure S12. ^{13}C NMR Spectrum (75 MHz) of Saccharomonopyrone C (**3**) in DMSO-d_6

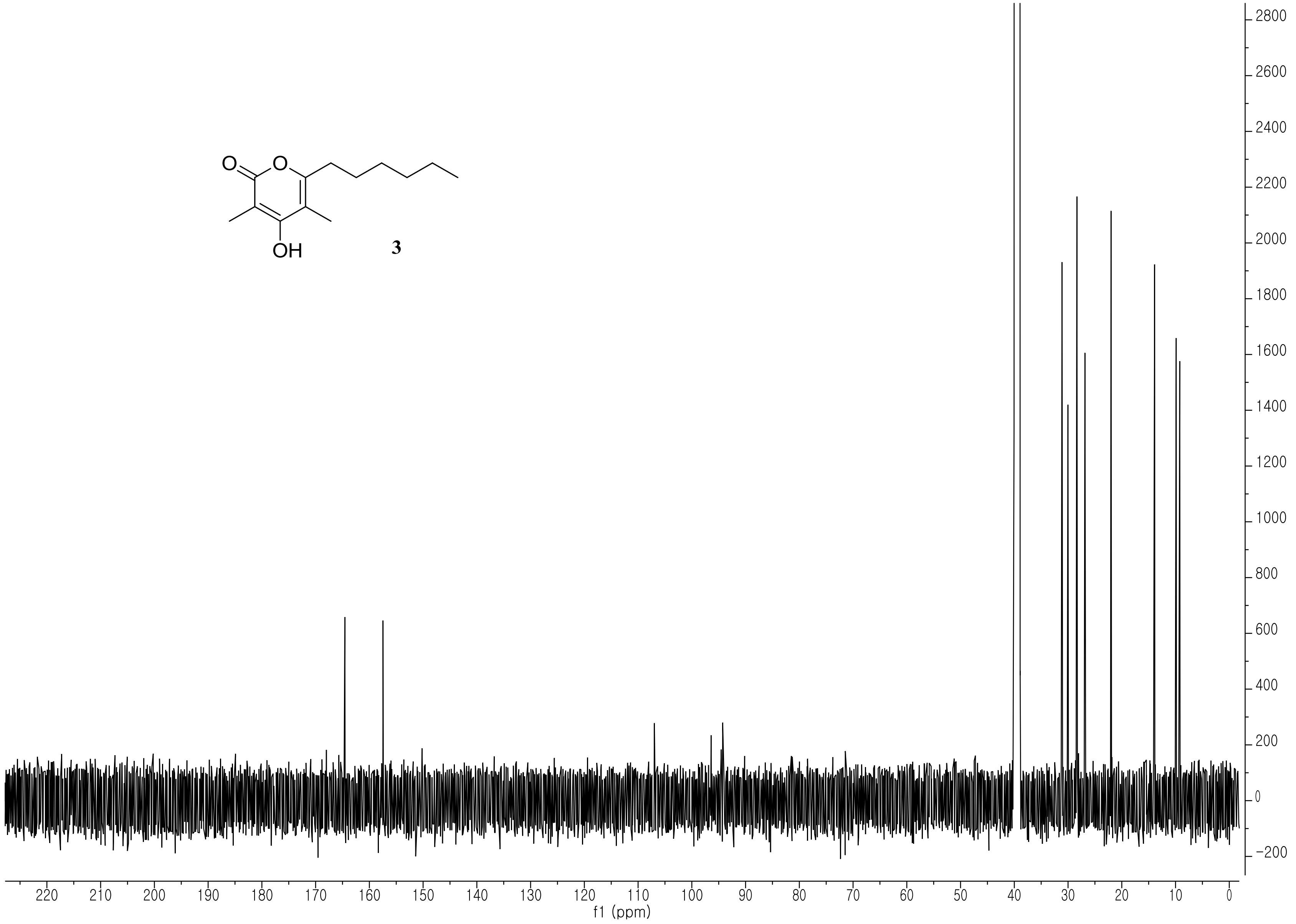

Figure S13. gCOSY Spectra (500 MHz) of Saccharomonopyrone A (**3**) in DMSO-d_6

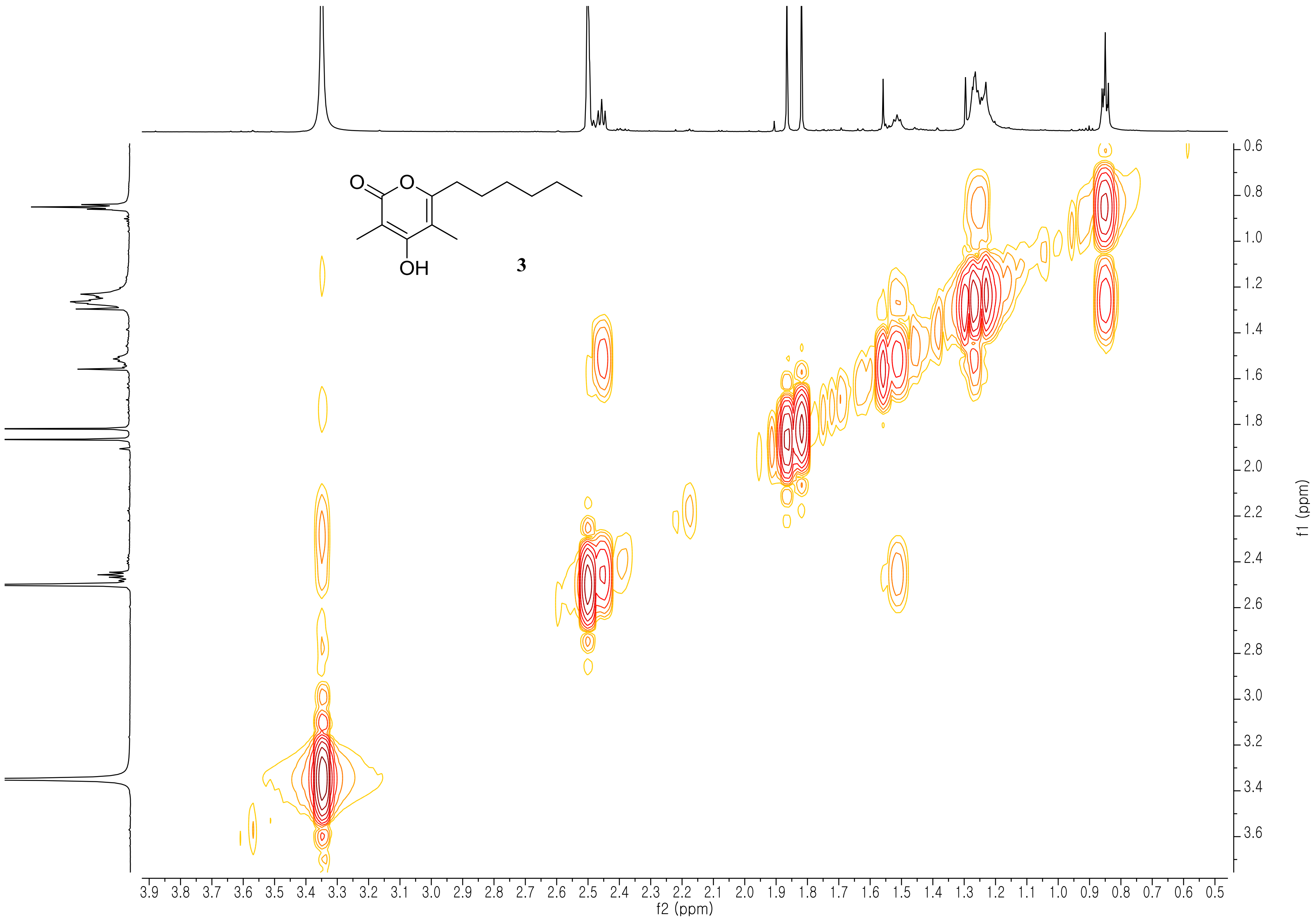

Figure S14. gHSQC Spectra (500 MHz) of Saccharomonopyrone A (**3**) in DMSO-d_6

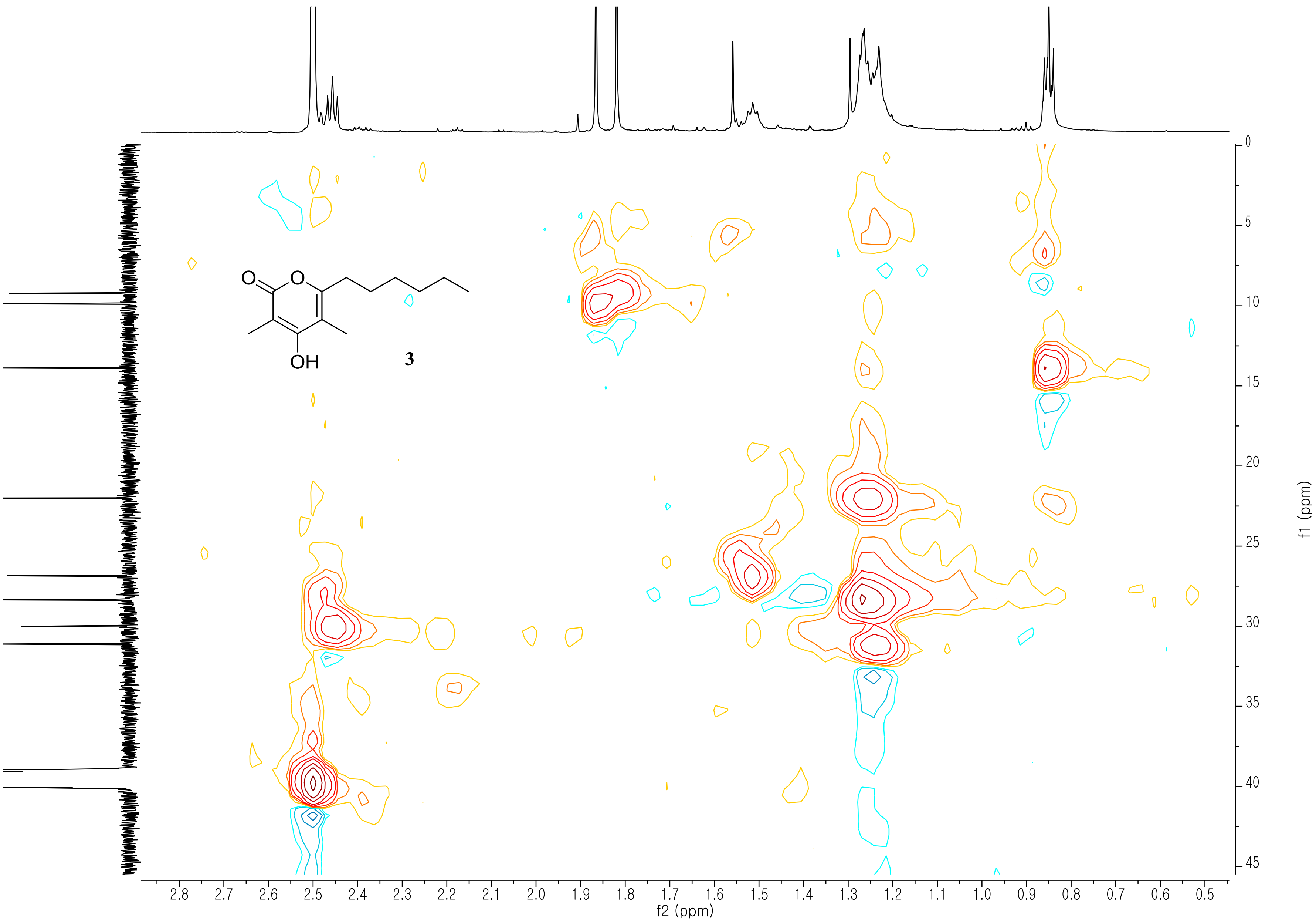

Figure S15. gHMBC Spectra (500 MHz) of Saccharomonopyrone A (**3**) in DMSO-d_6

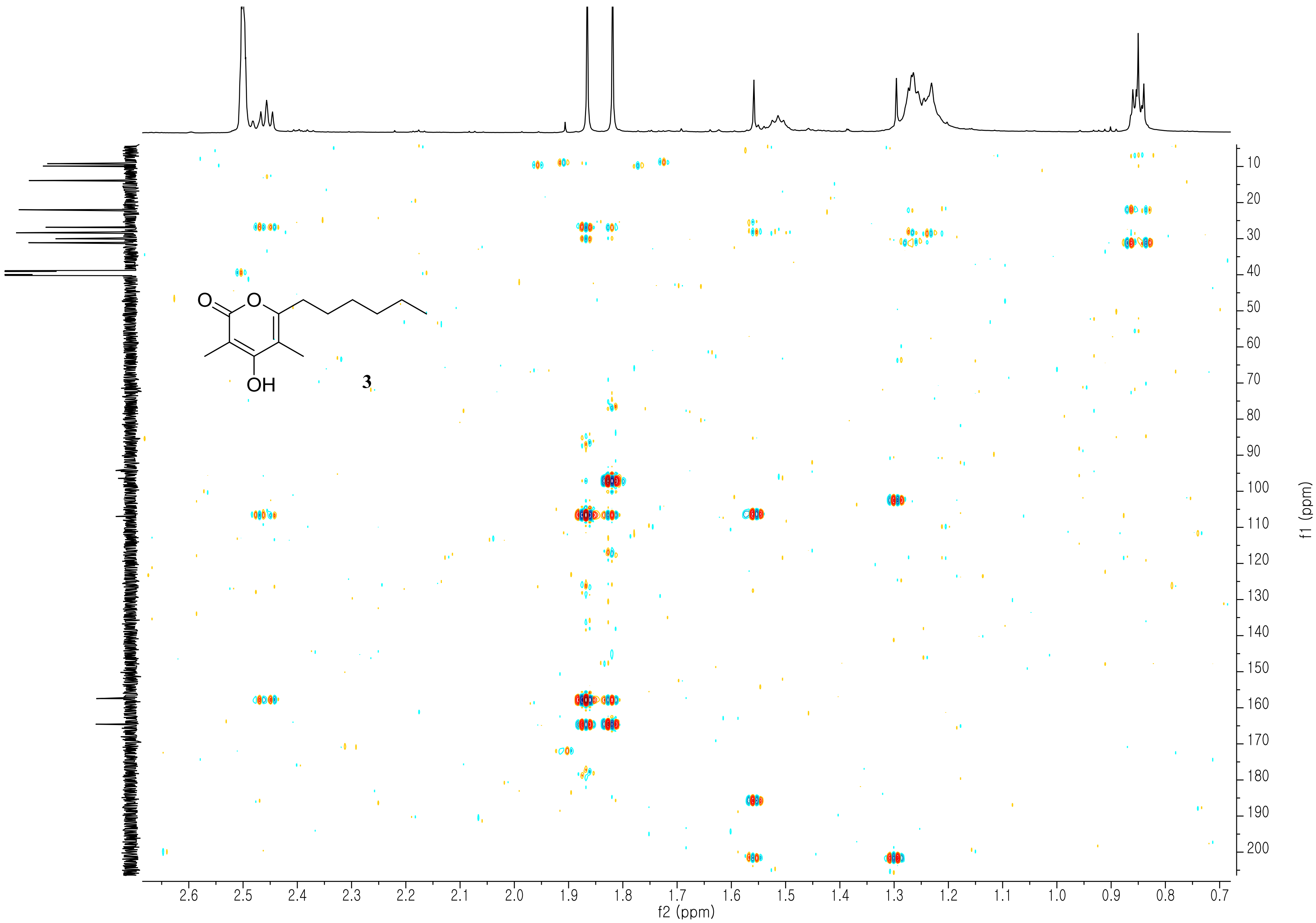

Figure S16. UV spectra of Saccharomonopyrones.

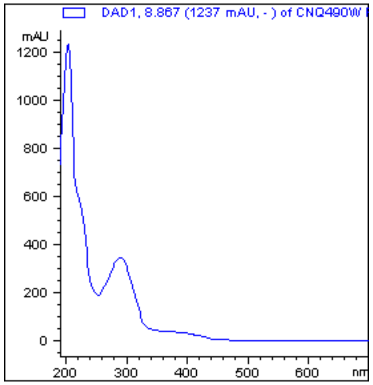

Saccharomonopyrone A

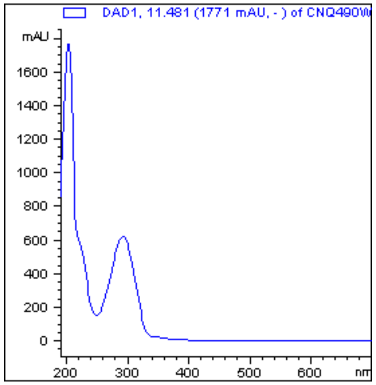

Saccharomonopyrone B

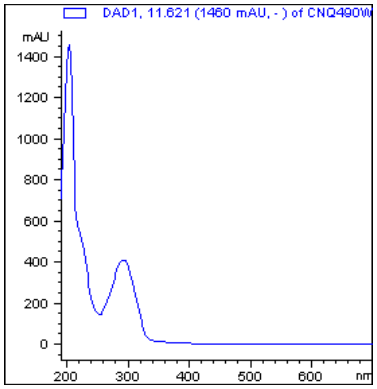

Saccharomonopyrone C
